# Validation of the Korean version of the Perinatal Infant Care Social Support scale: a methodological study

**DOI:** 10.4069/kjwhn.2021.12.12.1

**Published:** 2021-12-24

**Authors:** Mihyeon Park, Hyeji Yoo, Sukhee Ahn

**Affiliations:** College of Nursing, Chungnam National University, Daejeon, Korea

**Keywords:** Infant care, Mothers, Social support, Validation study

## Abstract

**Purpose:**

The purpose of this study was to develop and test the validity and reliability of the Korean version of the Perinatal Infant Care Social Support (K-PICSS) for postpartum mothers.

**Methods:**

This study used a cross-sectional design. The K-PICSS was developed through forward-backward translation. Online survey data were collected from 284 Korean mothers with infants 1-2 months of age. The 19-item K-PICSS consists of functional and structural domains. The functional domain of social support measures infant care practices of postpartum mothers. Exploratory factor analysis (EFA) and known-group comparison were used to verify the construct validity of the K-PICSS. Social support and postpartum depression were also measured to test criterion validity. Psychometric testing was not applicable to the structural social support domain.

**Results:**

The average age of mothers was 32.76±3.34 years, and they had been married for 38.45±29.48 months. Construct validity was supported by the results of EFA, which confirmed a three-factor structure of the scale (informational support, supporting presence, and practical support). Significant correlations of the K-PICSS with social support (r=.71, *p*<.001) and depression (r=–.40, *p*<.001) were found. The K-PICSS showed reliable internal consistency, with Cronbach’s α values of .90 overall and .82–.83 in the three subscales. The vast majority of respondents reported that their husband or their parents were their main sources of support for infant care.

**Conclusion:**

This study demonstrates that the K-PICSS has satisfactory construct validity and reliability to measure infant care social support in Korea.

## Introduction

The early stages following childbirth involve postpartum care for mothers’ physical recovery, as well as the process of acquiring parental roles and converting to a different family structure [1]. Mercer [2] proposed adopting “becoming a mother” as a process-focused phrase and retiring the phrase “maternal role attainment,” and suggested that social support serves as an important resource to facilitate the transition to parenthood. The social support necessary for mothers is defined as the actual amount of help provided for support needs, the degree to which they are satisfied with the help, and the number of people or support resources providing help [2]. Structural social support refers to a social network that can meet the mother’s support needs, consisting of an official network including healthcare providers and an informal network including the mother’s husband, family, and friends [3]. The existence and size of the network are also important because mothers can increase the quantity and quality of social support by utilizing these social networks [4]. Functional social support refers to the amount of support for each function of social support (e.g., direct help, provision of necessary information, emotional empathy, and positive evaluation) required by mothers to take care of the infant [4,5]. Recent studies have measured social support from structural and functional perspectives by evaluating how much help mothers receive from social networks and how satisfied they are [6-8].

Appropriate social support for mothers helps them to acquire new skills related to newborn caring behavior and promotes their physical and emotional recovery, thereby encouraging postpartum maternal health promotion behavior [2,9]. However, the expansion of nuclear families has often led to a lack of social support necessary for infant care, consequently increasing parenting stress and the risk of postpartum depression, which may cause difficulty in acquiring maternal roles or adapting to motherhood [10-12]. In addition, sufficient postpartum social support is a protective factor against postpartum depression, as an inadequate amount of social support and lack of support resources increase the incidence of postpartum depression [13-15]. It was found that higher levels of emotional support and practical social support from the husband were associated with fewer postpartum depression symptoms in mothers [5]. Therefore, nurses should guide mothers to assess the degree of practical help and available resources needed for infant care and to utilize available support resources [1,16].

Based on social exchange theory [17] and social support theory [18], Leahy-Warren et al. [4] developed the Perinatal Infant Care Social Support (PICSS) tool to assess the support needed for infant care, presenting the need for measurement according to the resources and types of social support suitable for the mother’s situation. The PICSS measures the type and amount of social support required for infant care, specifically in areas of functional social support (including supporting presence and practical support) and structural support resources [4].

The literature evaluating mothers’ social support notes that rather than structural support, multi-faceted social support tools have mainly evaluated functional support types [4,5,11,19,20]. However, few studies have focused on infant care in the postpartum adaptation process and evaluated both satisfaction with the actual support needs for parenting and utilization of support resources. Most Korean studies considering structural support resources dealt with the husband’s emotional and physical support [10] or physical, psychological, interpersonal, and informational support [10,21]; or else grouped the husband and others as support resources to measure emotional, informational, material, and evaluative support [11] or support for housework and infant care [22]. In addition, working women raising infants were most satisfied with the parenting of their mothers, relative to other caregivers, and received a large amount of practical support, especially in relation to raising the infant [22]. International studies also reported that husbands and family were commonly expected to be a resource, and that their practical support was most important [23]. Furthermore, high levels of satisfaction with information and emotional support through the internet and SNS-based communities were reported [24-26]. As such, the forms of support provided according to mothers’ requests appear to be similar in Korean mothers and those from other nations, but no tool focusing on support requests related to infant care has yet been used in studies in Korea. In addition, no studies have yet measured both structural and functional support needs. Thus, there is a need to measure the social support necessary for mother to provide infant care, and a tool developed for these purposes could assess support resources, support needs, and the degree of support, which are all necessary for the activation of social support.

However, regarding the social support necessary for mothers to provide infant care, careful consideration of cultural differences between Western and Korean culture is essential for applying a tool to research and practice, after confirming its suitability. Therefore, this study aimed to verify the reliability and validity of the Korean version of the PICSS (K-PICSS). The reliability analysis included item analysis and reliability coefficients, and the validity verification included construct validity using factor analysis and known-group comparisons, as well as criterion validity through a correlation analysis between related variables. As social support and supportive resources for infant care have been pointed out as facilitating factors that promote maternal role adaptation in Korean mothers [1], this instrument would be beneficial.

The purpose of this study was to translate the PICSS tool, which measures the support necessary for postpartum infant care and was developed by Leahy-Warren et al. [4], and to verify the validity and reliability of the Korean version.

## Methods

Ethics statement: This study was approved by the Institutional Review Board of Chungnam National University (202007-SB-096-01). Informed consent was obtained from the participants.

### Study design

**Figure 1.**
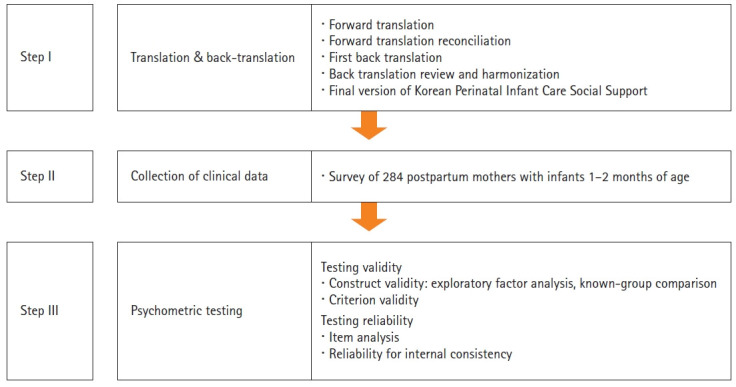
Korean version of Perinatal Infant Care Social Support development; a step-by-step approach.

This methodological study was conducted to translate the PICSS into Korean and verify its validity and reliability (Figure 1).

### Participants

The participants of this study were Korean mothers with infants, within 1 to 2 months of childbirth, who understood the purpose and method of this study and voluntarily agreed to participate. Participants were recruited by posting announcements on an online maternal/parenting community. Participants were recruited considering that the number of samples required for exploratory factor analysis is 10 times the number of variables [27], or at least 300 participants are required for sufficient feasibility testing of the instrument [28].

### Instruments

#### Social support for infant care

The research team obtained permission from the developer of the PICSS for translation into Korean. A four-stage process of translation was carried out, following the World Health Organization procedures for translation and back translation [29]. In the first stage, the original tool was translated into Korean by a doctorally trained nurse who can speak Korean and English. Concise and clear words and sentences that were easy for the general public to understand were chosen, avoiding direct translation. In the second stage, two professors specializing in women’s health nursing who were comfortable in both languages acted as an expert panel. They compared inconsistencies between the original text and translation and expressions due to cultural differences and suggested alternative words and expressions. In the third stage, the translated version was translated independently into English again by an English literature major fluent in English and Korean. In step 4, the researcher and the translator compared the translated tool with the original tool and confirmed the equivalence of each item. There were no major cultural differences in the translation process. One minor revision involved question 1, which was revised from “I can get information about taking care of my body after childbirth” to “I can get information about postpartum care” with consideration of the postpartum care culture in Korea. Finally, after checking for differences in meaning delivery in each question in consideration of the cultural, linguistic, and contextual aspects of the translation, the content validity of the translation was confirmed.

The 19-item PICSS measures the functional and structural aspects of the mother’s infant rearing. In terms of functionality, it consists of two subdomains: supporting presence (nine items) and practical support (10 items), with each item rated on a 5-point Likert (1, “not at all” to 5, “always”). A higher summed score (possible range, 19–95) indicates a higher level of social support. Validity was confirmed at the time of development, and the reliability of each subdomain at that time was good: Cronbach’s α was .90 for supporting presence and .86 for practical support [4]. Structural support measures specifically what kind of support the mother receives from official resources (nurse/midwives, doctors) and informal resources (husband, in-laws, parents, sisters, friends, and neighbors). Structural support (resource) for each question is reported as frequency and percentage.

#### Social support

To evaluate criterion validity, Park’s social support scale [19], which was used to measure mother’s social support [11,20] was adopted. This 25-item scale measures the subjective aspects of support to determine how an individual perceives and evaluates the actual quality of interpersonal relationships. The support type consists of four subfactors: emotional, material, informational, and evaluative support. Responses are rated on a 5-point Likert (1, “not at all” to 5, “always”) and summed (possible range, 25–125). At the time of development, the reliability and validity of the tool were confirmed [19] and reliability was reported as high (Cronbach’s α=.94), with good reliability for each subarea: emotional support, .87; material support, .84; information support, .85; and evaluation support, .83 [30]. In this study, Cronbach’s α was .94.

#### Postpartum depression

Postpartum depression was adopted as a concept suitable for criterion validity verification based on a study [15] showing a negative correlation with social support related to postpartum child-rearing. The Edinburgh Postnatal Depression Scale [31], which was adapted by Kim et al. [32], was used. The 10 self-reporting questions assess depression, anxiety, fear, guilt, and thoughts of self-harm on a 4-point Likert scale ranging from 0 to 3, with higher scores indicating postpartum depressed mood. Cronbach’s α was .87 at the time of development [31], .85 in a prior study of Korean mothers [32], and .86 in this study.

#### Data collection

Data collection was conducted from October 30 to December 30, 2020, using an online survey (Google Survey). After joining a maternal/parenting online community, the research team contacted the administrator, obtained permission to post a notice advertising the study, and invited participants from the newborn parenting group. Mothers interested in the study were guided to access the link to the informed consent document and online questionnaire. Over 2 months, a total of 302 mothers responded to the online survey. After excluding 18 responses (6.0%) that were partially completed or had insufficient responses, 284 responses (94.0%) were finally included in the analysis.

### Data analysis

The collected data were analyzed using IBM SPSS ver. 26.0 (IBM Corp., Armonk, NY, USA), and the statistical significance level was set to .05. The general characteristics and childbirth-related characteristics of mothers were analyzed by frequency analysis and descriptive statistics. The construct validity of the tool was confirmed by exploratory factor analysis and known-group comparison. For exploratory factor analysis, the maximum likelihood method and direct oblimin rotation were used, as one of several factor rotation methods. The factor extraction criterion was an eigenvalue of 1.0 or higher on a scree plot, and the factor loading criteria were a loading value above 0.3 for the primary factor and a difference of 0.2 between loadings when a variable was found to have two or more factors. In the group comparison method, known-group validity was confirmed using a one-way analysis of variance. Reference validity was analyzed using Pearson correlation coefficients to evaluate the relationship between social support and postpartum depression, which are reference variables related to the social support necessary for postpartum baby care. For reliability, item analysis and Cronbach’s α were used to test internal consistency. Frequency analysis was also conducted to investigate the utilization of structural support resources according to functional support items.

## Results

### General characteristics of the participants

The average age of mothers was 32.76 (standard deviation [SD], 3.34) years old, most mothers (95.1%) had an educational background above college graduation, and more than half (54.6%) were housewives. More than 70% of the participants had a monthly income of more than 4 million Korean won (corresponding to roughly 3,600 US dollars), and had been married for an average of 38.45 (SD, 29.48) months. More than half had given birth by cesarean section (50.4%), had male infants (54.9%), and answered that their subjective health status was good (55.2%). Most mothers (92.6%) answered that their infant was in good health (Table 1).

#### Exploratory factor analysis

Exploratory factor analysis was conducted on the 19 questions of the K-PICSS. The correlation coefficients between questions ranged from .16 to .57, and more than half of the variables used to perform factor analysis met the criterion that the correlation coefficient should exceed .30 [27]. In the sample suitability test, the Kaiser-Meyer-Olkin (KMO) value was .91, and the sphericity test of Bartlett yielded a result of χ2=2,262.97 (*p*<.001), indicating that the factor analysis was appropriate by establishing significance in the sphericity test and a KMO result above .90. The cumulative variance was 54.7%, which met the standard of 50% or more. As for the number of factors, three factors were extracted using the criterion of an eigenvalue of 1.0 or more on a scree plot. The explanatory power and question composition for each factor consisted of 37.3% for factor 1 (six items), 11.3% for factor 2 (seven items), and 6.1% for factor 3 (six items). The factor loading represents the degree of correlation between the item and the factor, and the factor loading of all items was .30 or more, satisfying the criteria (Table 2).

The attributes of the three factors identified through factor analysis of the K-PICSS were identified and named. Factor 1 was named “informational support” because it included advice or support for knowledge on solutions to parenting-related problems or on how mothers perform new roles in caring for infants (e.g., bathing, breastfeeding, and baby soothing). Factor 2 was named “supporting presence” because its items dealt with the existence of factors related to mothers’ acceptance of their needs, such as receptiveness to expressions of mothers’ physical and emotional discomfort, acknowledgment of mothers’ efforts to perform maternal roles, and the ability to help. Factor 3, which was named “practical support,” included tasks necessary for infant care (e.g., bathing, breastfeeding, baby soothing, changing clothes), and support for chores and caring for the baby.

#### Criterion validity

In order to test the criterion validity of the K-PICSS, the associations among K-PICSS, social support, and postpartum depression were evaluated. This tool showed a high level of positive correlation between K-PICSS and social support (r=.71, *p*<.001) and a significant positive correlation with social support in all subfactors of the K-PICSS (r=.46–.79, *p*<.001). In contrast, the K-PICSS showed a moderate negative correlation with postpartum depression (r=–.40, *p*<.001), and there was a significant negative correlation between postpartum depression and all subfactors of the K-PICSS (r=–.27 to –.43, *p*<.001) (Table 3).

#### Known-group comparison

In order to confirm construct validity through the known-group comparison method, differences in scores of the K-PICSS according to the spouse’s participation in childcare and housework were tested [33]. The score for social support required for infant care was higher when the husband’s participation in childcare was adequate (F=17.58, *p*<.001), and the division of housework with the husband was sufficient (F=16.38, *p*<.001). The validity of the group comparison was confirmed (Table 4).

### Item analysis and reliability verification

The correlation between each item and the factor was r=.30 or higher, verifying that the items were related to each other and that all items were properly composed. The K-PICSS average score was 23.51 (SD, 0.29), with average scores for the subdomains as follows: 21.20 (SD, 0.29) for informational support (six items), 27.93 (SD, 0.29) for supporting presence (seven items), and 21.40 (SD, 0.30) for practical support (six items).

For internal reliability, Cronbach’s α was .90 for the K-PICSS. In addition, each subdomain also had good internal reliability, as Cronbach’s α was .83 for informational support, .83 for supporting presence, and .82 for practical support (Table 2).

### Supporting resources for each item of the Korean version of Perinatal Infant Care Social Support

The results on utilization of support resources for each K-PICSS question showed that the most common source was parents (33.1%), followed by friends (27.6%), sisters (14.6%), and their husbands (8.2%). For information support on child-rearing, friends most often played the role of support resources (30.6%), whereas the husband was noted as the highest support resource in all items of supporting presence, as well as in most items of practical support. However, for help with breastfeeding in the practical support factor, parents (40.8%) were noted as the most common support resource (Table 5).

## Discussion

This study translated the PICSS into Korean to measure the level of social support necessary for mothers’ infant care and tested its reliability and validity in a sample of mothers caring for infants 1 to 2 months of age. The findings confirmed that the K-PICSS is a reliable and valid tool to measure social support required for infant care in Korea..

In this study, the tool translation guidelines were faithfully followed to secure the content validity of the original tool, and the linguistic and contextual meanings were reviewed with maternity nurses to facilitate an understanding of the items for cultural adaptation and cross-application. The items were finalized through a process of reviewing the words used in identifying maternal needs [34], so that the corresponding items in each subfactor showed consistent properties.

The construct validity of the tool was tested through exploratory factor analysis and the group comparison method. First, exploratory factor analysis confirmed that functional social support had a three-factor solution: practical support, informational support, and supporting presence. This is similar to the desire for sharing information, psychological support, and experiences—aside from more practical support—that was expressed in a qualitative study of mothers within 1 year of childbirth [34]. The original tool has two subdomains, practical support and supporting presence, and the same names were given in the K-PICSS because they corresponded to two of the three subdomains extracted in this study.

However, unlike the composition of the original tool, in the K-PICSS, the practical support subdomain was divided into informational support and practical support. This is different from the findings of validity testing of the Spanish version of the PICSS [35], which identified existence of support (emotional and evaluative support) and practical support (informative and instrumental support). This variation suggests that different types of support may have stemmed from differences in participants’ sociocultural support and resources related to childbirth and the postpartum period.

Practical support areas involve the help directly needed for infant care, including housework, breastfeeding, soothing, dressing, and bathing the infant. A prior study of working women in Korea noted that they were most satisfied with the parenting provided by their own mothers and received practical support related to infant care from their mothers [22]. This also parallels a study from the United States that reported that the husband and family members were expected to serve as reliable resources, and that their practical support was the most important [23].

The information support area encompasses information on childcare and postpartum care and includes questions related to soothing, changing clothes, and bathing the infant, as well as breastfeeding, child-rearing information, and postpartum care information. Mothers in Korea often find it difficult to immediately find a direct helper, role model or advisor to obtain extra hands-on help, learning, and advice on postpartum care and parenting due to changes in the social environment and family structure, leading to an increased predominance of nuclear families [36]. As such, the demand for information-related support seems to have been remarkably high. This may be related to embarrassment and anxiety due to the lack of information necessary for maternal role performance in the early stages of childbirth, a time when informational support needs are high [37]. It also supports a prior study that emphasized that support for childcare-related knowledge and information should be provided whenever necessary [38]. In recent years, demands for informational support through the internet and mobile services have been expressed, and mothers are reporting satisfaction with informational needs through answers to questions, advice, or knowledge transfer [24-26].

Supporting presence refers to whether there is someone to provide emotional and evaluative support such as caring, consoling, and sharing advice and experiences. Unlike a previous study [22] where social support was defined in terms of how much support (the amount of support resources) is given to the mother’s needs, this study focused on the presence of someone providing support when there is a demand for support. In particular, in the presence of supportive resources that can meet mothers’ needs for activities related to infant care itself, it is necessary to recognize whether mothers are receiving social support [39]. Therefore, when evaluating the social support necessary for infant care, this finding suggests the importance of evaluating the presence of support in addition to the practical or informational support required by the mother.

In terms of structural support, the frequency of resource utilization was confirmed by responses that allowed multiple choices of the eight available support resources for each area. Husbands appeared as the most commonly recognized support resource in the area of supporting presence, while women’s parents appeared as the highest support resource in most questions in the area of information support, consistent with findings of the original tool [4]. In the area of supporting presence and actual support demand, the most commonly utilized support resource was the husband first, followed by parents. This is consistent with previous studies [23,40] that showed that with the nuclear family system in modern society, husbands are tangible resources who help mothers manage postpartum care and participate in childcare.

The K-PICSS showed a high correlation with the social support tool [19], which is widely used in Korea. Since it consists of 19 items, it is a simpler alternative to the 25-item social support scale of Park [19]. The K-PICSS showed a moderate negative correlation with postpartum depression scores, which is in line with previous studies that reported high-level social support as a protective factor for postpartum depression, whereas lack of social support was a risk factor [13-15,40], thereby confirming criterion validity. As such, nurses can use the K-PICSS to assess the demand for social support for caring for infants in the early stages of parenting and plan nursing interventions to meet parents’ support needs. Nurses can provide practical education on postpartum care and practice to the mother and family upon discharge from the hospital or at a postpartum care center and can introduce relevant child-rearing-related social network services to meet the information needs [41].

Known-group validity is a type of evidence used to detect differences in outcome variables between known independent groups. In this study, mothers felt higher levels of social support in the presence of spousal participation in childcare and housework. This is consistent with a study finding that greater spousal support for infant care and household chores was associated with a higher level of social support [33].

A limitation of this study is that items on structural support resources did not contain online resources, so they do not reflect online resources that can be used for informational support. In Korea, online parenting communities are a popular source of information, easily accessed through the internet or social network services using smartphones, which may also make it possible to solve problems in real time through interactive communications if necessary [38]. In future studies, adding items to evaluate internet or mobile services as structural support resources may be beneficial.

In conclusion, the Korean version of the PICSS was confirmed to be a reliable and valid tool to assess the social support required for infant care, with specific factors including informational support, supporting presence, and practical support. The K-PICSS can be used in research and practice in Korea to identify the causes for a lack of social support and to suggest intervention directions.

## Figures and Tables

**Table 1.** General characteristics of participants (N=284)

Variable	Categories	n (%)	Mean±SD (range)
Age (year)	25–35	221 (77.8)	32.76±3.34 (25–43)
	36–43	63 (22.2)	
Education	High school	14 (4.9)	
	College	270 (95.1)	
Occupation	Yes	129 (45.4)	
	No	155 (54.6)	
Monthly income (KRW)	<4 million	82 (28.9)	
	≥4 million	202 (71.1)	
Marital period (month)			38.45±29.48 (6–120)
Type of birth	Vaginal birth	141 (49.6)	
	Cesarean birth	143 (50.4)	
Infant’s sex	Male	156 (54.9)	
	Female	128 (45.1)	
Maternal health status	Good	157 (55.2)	
	Poor	127 (44.8)	
Infant’s health status	Good	263 (92.6)	
	Poor	21 (7.4)	

KRW: Korean won (1 million KRW is approximately 900 US dollars).

**Table 2.** Validity and reliability testing of the Korean version of Perinatal Infant Care Social Support scale (N=284)

Factor	Item	Factor loading		
		F1	F2	F3
Informational support	I can get information on taking care of my body after birth.	.72		
	I can get information on infant changing/dressing.	.66		
	I can get consistent information on infant care.	.65		
	I can get information on infant comfort/settling.	.63		
	I can get information on infant feeding.	.61		
	I can get information on infant bathing.	.60		
Supporting presence	I have someone to talk to and share experiences with.		.79	
	I have someone to care for and comfort me.		.70	
	I have people to count on when things go wrong.		.68	
	I have someone to talk to about how I feel.		.64	
	If I need advice there is someone who will assist me.		.62	
	I have someone who shows me appreciation.		.48	
	Those close to me understand that it is ok for me to need help.		.46	
Practical support	I won’t be on my own taking care of my infant.			.64
	I have someone to help with routine housework.			.57
	I can get hands-on help with infant feeding.			.53
	I can get hands-on help with comforting my infant.			.52
	I can get hands-on help with infant changing/dressing.			.50
	I can get hands-on help with infant bathing.			.34
Eigenvalue		7.1	2.15	1.16
Explained variance (%)		37.37	11.3	6.11
Range of corrected item-total correlation		.52–.66	.46–.70	.52–.67
Cronbach’s α		.83	.83	.82

**Table 3.** Relationships among Korean version of Perinatal Infant Care Social Support, social support, and postpartum depression (N=284)

Variable	Korean Perinatal Infant Care Social Support, r (*p*)			
	Total	Informational support	Supporting presence	Practical support
Social support	.71 (<.001)	.46 (<.001)	.79 (<.001)	.54 (<.001)
Postpartum depression	–.40 (<.001)	–.27 (<.001)	–.43 (<.001)	–.31 (<.001)

**Table 4.** Comparisons of scores of the Korean version of the Perinatal Infant Care Social Support according to husband's involvement (N=284)

Variable	Categories	n (%)	Mean±SD	F (*p*)	Scheffé
Child-rearing involvement by fathers	Not adequate^a^	26 (9.2)	60.42±13.83	17.58 (<.001)	
	So-so^b^	59 (20.8)	66.91±9.79		a<b<c
	Adequate^c^	199 (70.1)	72.94±12.10		
Sharing household chores with husband	Not adequate^a^	42 (14.8)	61.45±11.50	16.38 (<.001)	
	So-so^b^	33 (11.6)	67.51±9.66		a<b, a<c
	Adequate^c^	209 (73.6)	72.85±12.13		

K-PICSS: Korean version of Perinatal Infant Care Social Support.

**Table 5.** Resources of social support based on mother’s support needs (N=284)

Factor	Item	n (%)							
		Husband	Parents-in-law	Parents	Sister	Friend	Neighbor	Doctor	Nurse
Informational support	I can get information on taking care of my body after birth.	11 (3.9)	8 (2.8)	100 (35.2)	31 (10.9)	75 (26.4)	23 (8.1)	23 (8.1)	13 (4.6)
	I can get information on infant changing/dressing.	27 (9.5)	9 (3.2)	103 (36.3)	46 (16.2)	75 (26.4)	19 (6.7)	3 (1.1)	2 (0.7)
	I can get consistent information on infant care.	19 (6.7)	4 (1.4)	82 (28.9)	43 (15.1)	87 (30.6)	28 (9.9)	18 (6.3)	3 (1.1)
	I can get information on how infant comfort/settling.	26 (9.2)	9 (3.2)	103 (36.3)	37 (13.0)	85 (29.9)	19 (6.7)	5 (1.8)	-
	I can get information on infant feeding.	21 (7.4)	4 (1.4)	85 (29.9)	44 (15.5)	81 (28.5)	23 (8.1)	11 (3.9)	15 (5.3)
	I can get information on infant bathing.	35 (12.3)	9 (3.2)	91 (32.0)	47 (16.5)	67 (23.6)	25 (8.8)	7 (2.5)	3 (1.1)
	Subtotal	139 (8.2)	43 (2.5)	564 (33.1)	248 (14.6)	470 (27.6)	137 (8.0)	67 (3.9)	36 (2.1)
Supporting presence	I have someone to talk to and share experiences with.	144 (50.7)	1 (0.4)	63 (22.2)	24 (8.5)	51 (18.0)	1 (0.4)	None	None
	I have someone to care and comfort me.	181 (63.7)	3 (1.1)	81 (28.5)	8 (2.8)	11 (3.9)	None	None	None
	I have people to count on when things go wrong.	204 (71.8)	1 (0.4)	61 (21.5)	4 (1.4)	13 (4.6)	1 (0.4)	None	None
	I have someone to talk to about how I feel.	174 (61.3)	1 (0.4)	30 (10.6)	24 (8.5)	55 (19.4)	None	None	None
	If I need advice there is someone who will assist me.	104 (36.6)	5 (1.8)	92 (32.4)	27 (9.5)	48 (16.9)	5 (1.8)	2 (0.7)	1 (0.4)
	I have someone who shows me appreciation.	212 (74.6)	13 (4.6)	17 (6.0)	12 (4.2)	29 (10.2)	1 (0.4)	None	None
	Those close to me understand that it is ok for me to need help.	146 (51.4)	3 (1.1)	68 (23.9)	22 (7.7)	44 (15.5)	1 (0.4)	None	None
	Subtotal	1,165 (58.6)	27 (1.4)	412 (20.7)	121 (6.1)	251 (12.6)	9 (0.5)	2 (0.1)	1 (0.1)
Practical support	I won’t be on my own taking care of my infant.	147 (51.8)	10 (3.5)	116 (40.8)	9 (3.2)	2 (0.7)	None	None	None
	I have someone to help with routine housework.	174 (61.3)	11 (3.9)	91 (32)	6 (2.1)	1 (0.4)	1 (0.4)	None	None
	I can get hands-on help with infant feeding.	84 (29.6)	3 (1.1)	116 (40.8)	14 (4.9)	31 (10.9)	13 (4.6)	7 (2.5)	16 (5.6)
	I can get hands-on help with comforting my infant.	123 (43.3)	13 (4.6)	111 (39.1)	14 (4.9)	16 (5.6)	6 (2.1)	None	1 (0.4)
	I can get hands-on help with infant changing/dressing.	128 (45.1)	13 (4.6)	109 (38.4)	10 (3.5)	17 (6.0)	5 (1.8)	None	2 (0.7)
	I can get hands-on help with infant bathing.	141 (49.6)	9 (3.2)	107 (37.7)	10 (3.5)	10 (3.5)	6 (2.1)	None	1 (0.4)
	Subtotal	797 (46.8)	59 (3.5)	650 (38.1)	63 (3.7)	77 (4.5)	31 (1.8)	7 (0.4)	20 (1.2)

## Supplementary materials

Further details on supplementary materials are presented online (available at https://doi.org/10.4069/kjwhn.2021.12.12.1).

Supplementary Table 1. 한국판 아기 돌봄에 필요한 사회적 지지 도구(Korean version of Perinatal Infant Care Social Support Scale [K-PICSS])
