# Supplementary material for: Validation of the Korean version of the Perinatal Infant Care Social Support scale: a methodological study
Source: Korean J Women Health Nurs. 2021 Dec 24;27(4):307–17. doi: 10.4069/kjwhn.2021.12.12.1 (PMC9328642; doi:10.4069/kjwhn.2021.12.12.1)
Supplement: Supplementary Table 1. — 한국판 아기 돌봄에 필요한 사회적 지지 도구(Korean version of Perinatal Infant Care Social Support Scale [K-PICSS]) [file kjwhn-2021-12-12-1suppl1.pdf]

Supplementary Table 1. 한국판 아기 돌봄에 필요한 사회적 지지 도구(Korean version of Perinatal Infant Care Social Support Scale [K-PICSS])

자신이 주위 사람들(가족, 친구, 그 외 사람들)로부터 얼마나 도움과 지지를 받고 있다고 느끼는가에 관한 질문입니다. 다음 문항을 읽고 자신이 느끼는 정도에 v 표시해 주시기 바랍니다.

번호	문항	전혀 그렇지 않다	그렇지 않다	보통이다	약간 그렇다	매우 그렇다
		1	2	3	4	5
1	산후 조리(몸 관리)에 대한 정보를 얻을 수 있다.					
2	아기 옷 갈아 입히기에 대한 정보를 얻을 수 있다.					
3	육아에 대한 일관된 정보를 얻을 수 있다.					
4	아기 달래기에 대한 정보를 얻을 수 있다.					
5	아기 수유에 대한 정보를 얻을 수 있다.					
6	아기 목욕에 대한 정보를 얻을 수 있다.					
7	나와 대화를 나누고 경험을 나눌 수 있는 사람이 있다.					
8	나를 돌보고 위로해 줄 사람이 있다.					
9	일이 잘못되어도 내가 의지할 사람이 있다.					
10	나의 감정에 대해 얘기할 사람이 있다.					
11	내가 조언이 필요하다면 나를 도와줄 사람이 있다.					
12	나에게 감사를 표현하는 사람이 있다.					
13	나의 주변 사람들은 내가 도움을 필요로 하는 것이 당연하다고 이해해 준다.					
14	나 혼자서 아기를 돌보지 않아도 된다.					
15	집안일을 도와줄 사람이 있다.					
16	아기 수유에 직접적인 도움을 받을 수 있다.					
17	아기를 달랠 때 직접적인 도움을 받을 수 있다.					
18	아기 옷을 갈아입힐 때 직접적인 도움을 받을 수 있다.					
19	아기를 목욕시킬 때 직접적인 도움을 받을 수 있다.					

♣ 당신은 도움과 지지가 필요할 때 누구에게(가족, 친구, 그 외 사람들) 도움을 받는지 v 표시해 주시기 바랍니다(중복선택 가능).

번호	문항	남편	시부모님	친정 부모님	자매	친구	이웃	의사	간호사
1	산후 조리(몸 관리)에 대한 정보를 얻을 수 있다.								
2	아기 옷 갈아입히기에 대한 정보를 얻을 수 있다.								
3	육아에 대한 일관된 정보를 얻을 수 있다.								
4	아기 달래기에 대한 정보를 얻을 수 있다.								
5	아기 수유에 대한 정보를 얻을 수 있다.								
6	아기 목욕에 대한 정보를 얻을 수 있다.								
7	나와 대화를 나누고 경험을 나눌 수 있는 사람이 있다.								
8	나를 돌보고 위로해 줄 사람이 있다.								
9	일이 잘못되어도 내가 의지할 사람이 있다.								
10	나의 감정에 대해 얘기할 사람이 있다.								
11	내가 조언이 필요하다면 나를 도와줄 사람이 있다.								
12	나에게 감사를 표현하는 사람이 있다.								
13	나의 주변 사람들은 내가 도움을 필요로 하는 것이 당연하다고 이해해 준다.								
14	나 혼자서 아기를 돌보지 않아도 된다.								
15	집안일을 도와줄 사람이 있다.								
16	아기 수유에 직접적인 도움을 받을 수 있다.								
17	아기를 달랠 때 직접적인 도움을 받을 수 있다.								
18	아기 옷을 갈아입힐 때 직접적인 도움을 받을 수 있다.								
19	아기를 목욕시킬 때 직접적인 도움을 받을 수 있다.								

https://doi.org/10.4069/kjwhn.2021.12.12.1
